# Compliance with behavioral guidelines for diet, physical activity and sedentary behaviors is related to insulin resistance among overweight and obese youth

**DOI:** 10.1186/1756-0500-4-29

**Published:** 2011-02-01

**Authors:** Jeannie S Huang, Michael Gottschalk, Gregory J Norman, Karen J Calfas, James F Sallis, Kevin Patrick

**Affiliations:** 1 Center for Wireless and Population Health Systems, Calit2 and Department of Pediatrics, University of California, San Diego and Rady Children's Hospital, 9500 Gilman Drive # 0811, La Jolla, CA 92093, USA; 2 Department of Pediatrics, University of California, San Diego and Rady Children's Hospital, 9500 Gilman Drive # 0984, La Jolla, CA 92093, USA; 3 Center for Wireless and Population Health Systems, Calit2 and Department of Family and Preventive Medicine, University of California, San Diego, 9500 Gilman Drive # 0811, La Jolla, CA 92093, USA; 4 Department of Family and Preventive Medicine, University of California, San Diego, 9500 Gilman Drive # 0067, La Jolla, CA 92093, USA; 5 Department of Psychology, San Diego State University, 3900 Fifth Avenue, Suite 310, San Diego, CA 92103, USA; 6 Center for Wireless and Population Health Systems, Calit2 and Department of Family and Preventive Medicine, University of California, San Diego, 9500 Gilman Drive # 0811, La Jolla, CA 92093, USA

## Abstract

**Background:**

Overweight and obesity are established risk factors for insulin resistance in youth. A number of behavioral recommendations have been publicized with the goal of improving glycemic control. However, there is limited information about whether meeting these behavioral recommendations actually reduces insulin resistance.

**Findings:**

92 youths 11 - 16 years with BMI ≥ 85% underwent oral glucose tolerance testing. HOMA-IR and AUC_Insulin_/AUC_Glucose _were calculated as measures of insulin resistance. Dietary and physical activity (PA) measures were performed. Assessments included whether or not participants met recommended levels of diet, PA and sedentary behaviors.

62% youths met criteria for insulin resistance. 82% (75/92) met at least one behavioral recommendation. Participants who met ≥ 1 dietary, sedentary, or PA recommendations had significantly reduced insulin resistance as compared with youth who did not. This relationship remained significant in multivariate modeling of insulin resistance adjusting for age, sex, and BMI.

**Conclusions:**

Even relatively minor behavior change may reduce insulin resistance in youth at risk for diabetes. Our findings support the relevance of current behavioral interventions for glycemic control.

**Trials Registration:**

Clinical Trials #NCT00412165.

## Background

Obesity is a well-known risk factor for the development of diabetes in childhood. Similarly, the effects of dietary, physical activity, and sedentary behaviors on insulin resistance have been well established. Dietary manipulation of macronutrients is important in the maintenance of glycemic control. Diet composition, specifically saturated fat and fiber, affects insulin resistance and risk of diabetes [1-3]. In prospective studies, improving physical activity improves insulin sensitivity [4,5]. Screen time and sedentary behaviors also are associated with abnormal glucose metabolism [6-8]. Guidelines have been developed for diet, physical activity (PA) and sedentary behaviors with the intent to improve glycemic control and prevent diabetes among children and adolescents. For example the American Diabetes Association (ADA) recommends a diet low in saturated fat and high in fiber with adequate carbohydrate intake, reduced sedentary behavior and increased physical activity [9]. However, the relationship between compliance with these behavioral recommendations and insulin resistance has not been well established. Thus, we evaluated these relationships in 92 overweight and obese children and adolescents.

## Methods

### Protocol

The study protocol (Clinical Trials # NCT00412165) was reviewed and approved by the Institutional Review Board at the University of California, San Diego. 92 overweight and obese (defined as BMI percentile for age and sex ≥ 85%) [10] children and adolescents aged 11 to 16 years voluntarily participated in a behavioral intervention aimed at reducing risk of diabetes. Participants were recruited from the pediatric endocrinology clinic at a metropolitan academic medical center and referring primary care offices. Inclusion criteria included: age 12 to 16 years; BMI ≥ 85% for age and sex; at least 2 ADA risk factors for diabetes such as family history of type 2 diabetes, race/ethnicity at risk, and/or signs of insulin resistance; ability to speak and read English; access to the internet; and planned residency in the local area for the upcoming year. Exclusion criteria included body weight > 285 pounds, residence in a foster care facility, pregnancy, current diabetes, and diagnosis with conditions (e.g. cardiovascular or musculoskeletal disease) that would limit ability to comply with physical activity recommendations. All participants provided written assent and their parents provided informed consent in accordance with university guidelines for human experimentation. For this report, only baseline data were analyzed.

### Measurements

The five behavioral recommendations assessed included: a) daily intake of ≥ 5 servings of fruits and vegetables [10,11]; b) daily moderate to vigorous physical activity for at least 1 hour [10,11]; c) screen time ≤ 2 hours daily [10]; d) saturated fat < 7% of total energy intake [11]; and e) 50-60% carbohydrates as a proportion of total energy intake [11]. Behavioral guidelines represent those recommended by the American Heart Association [11,12], American Diabetes Association [13,14], and the American Academy of Pediatrics [10] to improve glycemic control and reduce cardiovascular risk. Self-report surveys were utilized to determine whether or not participants met recommended levels of diet, physical activity and sedentary behaviors. Physical activity was assessed with the 7-Day Physical Activity Recall (PAR), one of the most widely studied self-report measures of physical activity [15]. Dietary intake nutrient estimates were derived from the Youth/Adolescent Questionnaire (YAQ), a validated self-administered food frequency questionnaire (FFQ) for adolescents [16,17]. For sedentary behaviors, participants completed a self-report measure of recent school day and non-school day time spent watching television, playing computer/video games, etc. [18]. Demographic data were also collected.

### Insulin resistance measurement

Insulin resistance was measured via oral glucose tolerance testing. Blood samples were collected at: 0, 10, 20, 30, 60, 90, and 120 minutes after a 12 hour fast and consumption of a 75 g standard glucose drink at the 0 time-point. Serum samples were analyzed for glucose and insulin using standard biochemical procedures. Total AUC (area under the curve)'s for both insulin and glucose were calculated using the trapezoidal method [19]. The integrated AUC_insulin_/AUC_glucose _ratio was then determined as a measure of insulin resistance [20,21] with elevated levels indicating higher insulin resistance. HOMA-IR was calculated as the fasting insulin level (μU/mL) × early morning fasting blood glucose level (mg/dL)/405. A HOMA-IR level > 3.16 was defined as insulin resistance [22].

### Statistical Methods

Baseline demographic characteristics were evaluated using usual distribution analyses. Univariate evaluations of insulin resistance measures according to adherence vs. non-adherence with each of the behavior recommendations and according to compliance with at least 1 behavioral recommendation v. no compliance were performed using the van der Waerden test for AUC_Insulin_/AUC_Glucose _(continuous variable) and Fisher's exact test for HOMA-IR (given its binomial distribution). Owing to relatively low compliance rates of specific behavior recommendations (presented in Table 1) and to improve interpretability of results, a summary measure of adherence (i.e. adherence to no vs. at least 1 behavior recommendation) was utilized for multivariate analyses. Multivariate analyses of insulin resistance (both AUCM_Insulin_/AUC_Glucose _and HOMA-IR) were performed, entering all variables with potential associations (defined as univariate relationships with p ≤ 0.25). Statistical analyses were performed on questionnaire responses using JMP 5.0 statistical software (Cary, NC). Significance for all analyses was set at p < 0.05.

**Table 1:** Percent of youth meeting behavioral recommendations. N = 92.

Recommendation	Adherence rate
Daily intake of ≥ 5 fruits and vegetables	18%

Moderate to vigorous physical activity for ≥ 1 hour daily	36%

Screen time ≤ 2 hours daily	17%

Total dietary carbohydrate proportion 50-60% of total energy intake	61%

Saturated fat intake < 7% of total energy intake	1%

## Results

The 92 evaluated children and adolescents had a mean age of 14 years ± 1.5 years (mean ± SD), 37% were male, and 73% were hispanic, 18% white, and 15% black. The mean BMI of the population was 33.8 ± 5.0 kg/m^2^, and 43% of the cohort had a BMI% > = 99%. Mean AUC_insulin_/AUC_glucose _was 1.14 ± 0.74, and insulin resistance was diagnosed based on the HOMA-IR criterion in 57 (62%) cohort participants. Level of adherence with each of the dietary, physical activity, and sedentary behavioral guidelines is reported in Table 1. In general, adherence to guidelines was suboptimal. 18% did not comply with any of the behavioral guidelines, and only 12% met 3 of the guidelines. No participant met 4 or more of the guidelines.

### Measures of insulin resistance compared to meeting behavioral recommendations

AUC_Insulin_/AUC_Glucose _was elevated among participants who did not meet any of the behavioral guidelines as compared to subjects who met at least one (1.04 ± 0.56 v. 1.58 ± 1.21, met at least one guideline (N = 75) vs. none (N = 17), p = 0.03). In multivariate modeling, the significant relationship between meeting at least one behavioral recommendation and insulin resistance (AUC_Insulin_/AUC_Glucose_) remained, adjusting for age, BMI, and sex (Table 2).

**Table 2:** Multivariate modeling of insulin resistance. N = 92.

AUC_Insulin_/AUC_Glucose_ Whole model p < 0.01. R^2 ^= 0.14.		
Variable	Estimate	p-value
Age (years)	- 0.09	0.09
BMI (kg/m^2^)	0.03	0.04
Sex (female)	- 0.008	0.92
Meets no behavioral guideline (yes)	0.27	0.006

**HOMA-IR > 3.16 (yes:no)** **Whole model p < 0.01. R^2 ^= 0.11.**		

Variable	Odds Ratio	
Age (years)	0.86	
BMI (kg/m^2^)	1.12	
Sex (female)	1.55	
Meets no behavioral guideline (yes:no)	5.38	

Similarly, the proportion of insulin resistance diagnosed by HOMA-IR was greater among participants who met no guidelines as compared to participants who met at least one dietary, physical activity, or sedentary guideline (56% vs. 88%, compliant with at least one guideline vs. none, p = 0.01). The multivariate model revealed meeting at least one behavioral guideline similarly remained a significant correlate with insulin resistance (p = 0.04) after adjusting for age, sex, and BMI.

Analysis of insulin resistance measures according to whether or not participants met specific behavioral recommendations (e.g. reducing screen time to < 2 hours daily) did not reveal any significant differences (all p-values from these evaluations > 0.05).

## Discussion

In this group of obese children and adolescents, meeting at least one of the recommended behavioral guidelines for diet, physical activity and sedentary behavior was associated with improved insulin resistance. No single behavioral guideline was associated with improved insulin resistance in all participants, but statistical power for these analyses was low. Our finding that meeting any behavioral guideline was related to lower risk of insulin resistance supports the validity of the recommendations for diabetes prevention and suggests that multiple behaviors need to be considered, since no single behavior played a dominant role.

In general, participants in this study did not meet guidelines for the target behaviors. Adherence rates of our cohort to evaluated behavioral guidelines are generally consistent with recent reviews of dietary guideline adherence among school children demonstrating poor compliance with dietary recommendations (i.e. ~20% consuming ≥ 5 fruits and vegetables daily) [23] and prior studies documenting 11-50% of youth meeting physical activity and sedentary behavioral recommendations [24,25].

Limitations of the present study include the issue of self-report measures for measured behaviors and the low rate of behavior guideline compliance in our cohort. In considering self-report as a measure of adherence to behavioral recommendations, reporting bias would have likely led to an overestimation of adherence as compared to actual rates. However, adherence rates reported by our cohort were consistent with actual observed rates of physical activity and sedentary habits published in other studies [24,25]. Our reported rates of dietary guideline compliance are likewise similar to those reported in much larger-scale studies [23], and all utilized measures have prior evidence of reliability and validity [15-18]. In regards to the poor compliance with behavior guidelines in our cohort, this is likely a function of the characteristics (i.e. weight status) of our recruited population. However, this low level of compliance did restrict our ability to determine whether compliance with specific behavior guidelines and/or multiple guidelines (especially compliance with > 3 guidelines) improve insulin sensitivity. Additional study with a population demonstrating greater behavioral compliance variability is thus needed. Lastly, the cross-sectional design makes it impossible to disentangle the complex relationships between the influence of meeting behavioral recommendations and insulin resistance. Evaluation of intervention effects from this behavioral trial at completion may provide additional information about causal effects of behavior change.

In summary, our findings support the relevance of current behavioral recommendations for glycemic control. In particular, our data suggests that even relatively minor impact (i.e. affecting only one behavioral goal) behavior change interventions may reduce risk for this important source of morbidity in children and adolescents.

## List Of Abbreviations

BMI: body mass index; AUC: area under the curve; HOMA-IR: homeostasis model assessment of insulin resistance; PA: physical activity.

## Competing interests

Karen Calfas, Kevin Patrick and James Sallis are co-founders of and receive income from SanTech, Inc. (La Jolla, Ca.), which is developing products related to the research described in this paper. The terms of this arrangement have been reviewed and approved by the University of California, San Diego and San Diego State University in accordance with their respective conflict of interest policies.

## Authors' contributions

JH performed the statistical analysis and drafted the manuscript. KP, MG, KC, JS, and GN conceived of the study, participated in its design and coordination, and helped to critically revise the manuscript. All authors read and approved the final manuscript.
